# Quantitative Videofluoroscopic Analysis of Swallowing Physiology and Function in Individuals With Chronic Obstructive Pulmonary Disease

**DOI:** 10.1044/2020_JSLHR-20-00154

**Published:** 2020-10-26

**Authors:** Renata Mancopes, Melanie Peladeau-Pigeon, Emily Barrett, Andrea Guran, Sana Smaoui, Adriane Schmidt Pasqualoto, Catriona M. Steele

**Affiliations:** a The KITE Research Institute, Toronto Rehabilitation Institute, University Health Network, Ontario, Canada; b Dysphagia Laboratory, Graduate Program of Human Communication Disorders, Federal University of Santa Maria, Rio Grande do Sul, Brazil; c Rehabilitation Sciences Institute, University of Toronto, Ontario, Canada

## Abstract

**Purpose:**

Dysphagia is a serious extra pulmonary manifestation of chronic obstructive pulmonary disease (COPD). However, the nature of abnormalities in swallowing physiology in COPD has yet to be clearly established. We explored the frequency of swallowing measures outside the healthy reference range in adults with COPD.

**Method:**

Participants were 28 adults aged 41–79 years (18 men, 20 women) with stable COPD. Disease severity was classified as GOLD (Global Initiative For Chronic Obstructive Lung Disease) Stages 1 (4%), 2 (25%), 3 (53%), and 4 (18%). Participants underwent a videofluoroscopy and swallowed 20% w/v thin barium in, followed by 20% w/v mildly, moderately, and extremely thick barium prepared with a xanthan gum thickener. Blinded duplicate ratings of swallowing safety, efficiency, kinematics, and timing were performed according to the ASPEKT method (Analysis of Swallowing Physiology: Events, Kinematics and Timing). Comparison data for healthy adults aged < 60 years were extracted from an existing data set. Chi-square and Fisher's exact tests compared the frequencies of measures falling < 1 *SD*/ > 1 *SD* from mean reference values (or < the first or > the third quartile for skewed parameters).

**Results:**

Participants with COPD did not display greater frequencies of penetration–aspiration, but they were significantly more likely (*p* < .05) to display incomplete laryngeal vestibule closure (LVC), longer time-to-LVC, and shorter LVC duration. They also displayed significantly higher frequencies of short upper esophageal sphincter opening, reduced pharyngeal constriction, and pharyngeal residue.

**Conclusion:**

This analysis reveals differences in swallowing physiology in patients with stable COPD characterized by impaired safety related to the mechanism, timing, and duration of LVC and by impaired swallowing efficiency with increased pharyngeal residue related to poor pharyngeal constriction.

Chronic obstructive pulmonary disease (COPD) represents an important public health concern, comprising the fourth most common cause of mortality worldwide, and is expected to become the third most common cause of mortality by the end of 2020 (Global Initiative For Chronic Obstructive Lung Disease [GOLD], 2020). COPD is characterized by persistent airflow obstruction with an inflammatory response and systemic manifestations (GOLD, 2020). Peripheral muscle dysfunction and dynamic respiratory hyperinflation lead to restrictions in activities of daily living. Dysphagia (swallowing impairment) is a serious extra pulmonary manifestation of COPD, which impacts both physical health and psychosocial well-being (Garand et al., 2018). Furthermore, dysphagia-related aspiration (i.e., entry of foreign material into the lower airway) can be a trigger for exacerbations of COPD (Steidl et al., 2015). One study of 78 male patients with COPD reported a dysphagia prevalence of 85%, with penetration–aspiration observed in 56% of subjects (Good-Fratturelli et al., 2000). Aspiration during swallowing in patients with COPD is attributed to altered thoracic and abdominal respiratory biomechanics, pulmonary hyperinflation, and discoordination between breathing and swallowing (Steidl et al., 2015).

Research regarding dysphagia in COPD suggests that risk factors for aspiration are commonly present (Cassiani et al., 2015; Chaves et al., 2014; Clayton et al., 2014; Macri et al., 2013; Steidl et al., 2015). For example, a recent study by Nagami et al. (2017) reported a 25.5% prevalence of discoordination between breathing and swallowing in a sample of 65 individuals with COPD. Instead of the typical exhalation–swallow–exhalation pattern, in which swallowing occurs partway through the expiratory phase of breathing (Martin-Harris et al., 2003), these individuals showed higher frequencies of inhalation preceding or immediately following the respiratory pause associated with swallowing. Postswallow inspiration is thought to increase the risk of aspiration, particularly during the middle of serial ingestion cycles or in situations where residual material is present in the pharynx near the entrance to the airway (Butler et al., 2007).

In addition to altered respiratory–swallow coordination patterns, differences in swallow timing measures have been reported in individuals with COPD, including prolonged pharyngeal transit time (Chaves et al., 2014), prolonged laryngeal vestibule closure (LVC), and prolonged duration of hyoid bone movement (Cassiani et al., 2015). Pharyngeal residue has also been reported as a feature of swallowing in COPD (Clayton et al., 2014; Macri et al., 2013). Atrophy and weakness of the respiratory muscles are commonly seen in COPD (Cassart et al., 1997), but whether COPD also leads to weakness in the swallowing musculature is unclear.

Although studies point to changes in the biomechanics of swallowing, there is no consensus regarding the prevalence or mechanisms of dysphagia in COPD. Chronic recruitment of the accessory muscles of respiration in pulmonary hyperinflation may serve as an antagonist factor that restricts hyolaryngeal movement (Steidl et al., 2015; Yawn, 2013). This may contribute to poor LVC, reduced upper esophageal sphincter (UES) opening, and pharyngeal residue, all of which increase the risk of penetration and aspiration (Cvejic et al., 2011; Park et al., 2010). In addition, reduced laryngopharyngeal sensitivity has been shown in people with COPD (Clayton et al., 2014).

A recent article by Garand et al. (2018) described swallowing function in a group of 10 underweight patients with severe but stable COPD (GOLD Stages 3 and 4). Study participants completed a videofluoroscopy following the MBSImP protocol (Martin-Harris et al., 2008). The analysis showed higher maximum scores on the Penetration–Aspiration Scale (PAS; Rosenbek et al., 1996) in the COPD patients (maximum PAS score: 8) compared to a group of 37 age- and sex-matched healthy controls (maximum PAS score: 5). Other aspects of swallowing that were reported to be impaired in 50% or more of the COPD cohort included MBSImP Components 2: tongue control during bolus hold (50%), 3: bolus preparation/mastication (70%), 5: oral residue (100%), 6: initiation of the pharyngeal swallow (100%), 7: soft palate elevation (50%), 11: LVC (50%), 14: pharyngoesophageal segment opening (90%), 15: tongue base retraction (100%), 16: pharyngeal residue (100%), and 17: esophageal clearance (100%). Surprisingly, however, impairment was also reported to be present in 50% or more of the 37 healthy control participants for several MBSImP components (oral residue, initiation of the pharyngeal swallow, laryngeal elevation, anterior hyoid excursion, UES opening, tongue base retraction, and pharyngeal residue). The high prevalence of abnormal findings in these healthy controls leads to an unclear picture of the nature of swallowing impairment in the COPD sample. The scoring approach of MBSImP identifies the worst ordinal scale score for each parameter across a range of swallowing tasks and therefore does not easily permit the identification of frequencies of or differences in swallowing dysfunction according to bolus consistency. Furthermore, the MBSImP rating protocol does not include timing measures of bolus flow, pharyngeal or laryngeal events.

Given the lack of clear findings regarding the pathophysiology of swallowing in COPD, we believe that further research is needed. The purpose of this study was to characterize swallowing function and physiology based on videofluoroscopy in patients with stable COPD, compared to recently published reference data for swallowing in healthy adults under the age of 60 years (Steele et al., 2019). Specifically, we were interested to determine how often measures of swallowing in COPD would fall more than 1 *SD* beyond the healthy reference mean or outside the healthy interquartile reference range. Values beyond the first and third quartile boundaries (henceforth Q1 and Q3, respectively) can be considered “at risk” or approaching abnormal (Ceriotti et al., 2009; Ozarda, 2016; Steele et al., 2020). If significantly higher frequencies of at-risk values for particular parameters are seen in patients with a given disease compared to healthy controls, this would point to patterns of pathophysiology that can be considered hallmark characteristics of swallowing impairment in that disease.

We hypothesized that individuals with COPD would display higher frequencies of at-risk values for measures of penetration–aspiration and LVC. Given evidence in the literature that individuals with COPD may experience muscle weakness, we also hypothesized that individuals with COPD might display higher frequencies of at-risk values for measures of swallow timing, peak hyoid position, pharyngeal constriction, pharyngeal area at rest, and pharyngeal residue. By following a standard protocol and comparing measures of swallowing physiology in COPD to reference values for healthy adults, our goal is to elucidate the nature of swallowing pathophysiology in COPD. Ultimately, we hope that this analysis of the mechanisms behind at-risk swallowing in individuals with COPD will inform treatment strategies and clinical decision making.

## Method

### Participants

A sample of adult patients was recruited from the pulmonology and physiotherapy outpatient clinic at an academic tertiary medical center in the Rio Grande do Sul State, Brazil, over a 2-year period from January 2017 to December 2018. The inclusion criteria required participants to be aged 40 years or older, to have a diagnosis of COPD according to the GOLD, and to be medically stable (without exacerbation of symptoms for at least the last 30 days). Individuals who were dependent on oxygen or already receiving treatment for management of dysphagia were excluded. We also excluded pregnant women and individuals with histories of stroke, brain injury, other neurological diseases, head and neck cancer, other lung diseases, history of prior surgery in the oral cavity and laryngopharyngeal region, or those using medications that could compromise level of awareness and the reliability of the evaluation.

The study sample included 28 adults (18 men and 10 women) with a diagnosis of COPD confirmed by spirometry. Demographic details are summarized in Table 1. The mean age of participants in the COPD cohort was 65 years (range: 41–79). As is conventional in respiratory medicine, the severity of COPD was classified by the GOLD staging method, with four possible degrees of severity from 1 (*mild*) to 4 (*very severe*), which capture the degree of airflow limitation, based on measures of forced expiratory volume, together with consideration of patient symptoms and risk of disease exacerbation (GOLD, 2020). None of the participants in the COPD cohort reported exacerbations in the 3 months prior to study participation. For the purposes of comparison, a set of control group data was extracted retrospectively from the data set previously collected by Steele et al. (2019), from which reference data for healthy adults under the age of 60 years have been published. The sample for that reference values study comprised 38 healthy participants (19 men, 19 women), with a mean age of 34 years (range: 21–58). In order to match the number of observations across cohorts, the control group data were limited to values for the first bolus of each task from the healthy reference data set. The study protocol received human subjects approval from the appropriate institutional review boards, and all participants provided written informed consent.

**Table 1.** Demographic characteristics of the study sample.

Parameter	COPD (*n* = 28)	Healthy control group (*n* = 38)
Age		
*M* ± *SD*	64.74 ± 8.29	34
Range	41–79	21–58
Sex (female)	10 (35.71%)	19 (50%)
Pulmonary function		
FEV1 % predicted	44.26 ± 17.56	
FVC % predicted	69.81 ± 22.41	
FEV1 %/FVC	48.4 ± 12.22	
GOLD frequency distribution		
GOLD 1	3.6% (1)	
GOLD 2	25% (7)	
GOLD 3	53.6% (15)	
GOLD 4	17.9% (5)	

*Note.* COPD = chronic obstructive pulmonary disease; FEV1 = forced expiratory volume in the first second; FVC = forced vital capacity; GOLD = Global Initiative for Chronic Obstructive Lung Disease.

### Data Collection

Videofluoroscopy data from the COPD cohort were collected using a Siemens AxionIconos R200 fluoroscope, operating in continuous fluoroscopy mode without magnification, and recorded with Zscan capture software Version 6 (Zscan; https://zscansoftware.com). Liquid stimuli were prepared with Bariogel diluted to a 20% weight-to-volume (w/v) concentration with water and thickened with a xanthan gum thickener (Resource ThickenUp Clear, Nestlé Health Science). Flow testing using the International Dysphagia Diet Standardisation Initiative (IDDSI) testing methods (Hanson et al., 2019) using a 10-ml syringe (Becton Dickinson Model BD 303134) confirmed liquid consistency with residual fluid column heights after 10 s of flow of 0, 7.0, 9.6, and 10 ml (i.e., no drip) for the thin, mildly thick, moderately thick, and extremely thick stimuli, respectively. Each participant swallowed two boluses of each stimulus. For the thin, mildly thick, and moderately thick stimuli, one bolus was taken by 10-ml spoon and one was taken by comfortable sip from a cup. Both boluses of extremely thick liquid were taken by 10-ml spoon. The instructions were to swallow naturally, without waiting for a cue from the investigator. In order to match administration methods for comparison with the healthy reference cohort, data for the analysis were limited to the cup-drinking task for the thin, mildly thick, and moderately thick liquids and to the first spoon-delivered bolus for the extremely thick liquid consistency.

### Frame Rate

Video image acquisition rate was confirmed using a method previously described by Peladeau-Pigeon and Steele (2015), in which the moving arm of a metronome was recorded under fluoroscopy at 40 and 44 beats per minute and with fluoroscopy settings of zero, 1× and 2× magnification. Subsequent frame-by-frame tracking of the metronome arm movement was performed using ImageJ software (National Institutes of Health), allowing extrapolation and plotting of the movement waveform over time and calculation of frame frequency. This method determined the COPD videofluoroscopy recordings to contain 36 frames per second, with no duplicate frames in the image stream. Based on this, timing measures were converted from frames to milliseconds at a rate of 27.7 ms/frame.

### Videofluoroscopy Rating

Each bolus-length videofluoroscopy recording was de-identified, stripped of audio, and randomized for blinded rating. Videofluoroscopy rating was conducted using ImageJ software according to the ASPEKT method (Analysis of Swallowing Physiology: Events, Kinematics and Timing; Steele et al., 2019). The parameters of interest are listed and defined in Table 2. Additional details regarding the definitions and measurement methods for these parameters can be found in the appendix of the healthy reference values article (Steele et al., 2019). Supplementary reference tables identifying reference boundaries both for normally distributed and skewed parameters are available, by bolus consistency, at https://steeleswallowinglab.ca/srrl/wp-content/uploads/ASPEKT-Method-Reference-Value-Tables-V1.3.pdf.

**Table 2.** Parameters measured in this study.

Parameter	Abbreviation	Unit of measurement	Definition
No. of swallows per bolus		Integer	
Penetration–Aspiration Scale	PAS	Nominal	8-point scale characterizing depth of and response to penetration–aspiration (Rosenbek et al., 1996); at-risk scores of 3 and higher
Swallow Reaction Time	SRT	ms	Time from bolus passing mandible to hyoid burst onset
Hyoid burst onset to upper esophageal sphincter opening	HYB-to-UESO	ms	Interval from hyoid burst onset (HYB) until upper esophageal sphincter opening
Upper esophageal shincter opening duration	UESO duration	ms	Interval from onset of UES opening until UES closure
Pharyngeal transit time	PTT	ms	Sum of SRT, HYB-to-UESO, and UESO duration intervals
Time-to-laryngeal vestibule closure	Time-to-LVC	ms	Interval from HYB until laryngeal vestibule closure. Also called “LVC reaction time” in previous studies (Steele et al., 2019; Humbert et al., 2018).
Laryngeal vestibule closure duration	LVC duration	ms	Interval from laryngeal vestibule closure until opening of the laryngeal vestibule
Integrity of laryngeal vestibule closure	LVC Integrity	Nominal	Degree of laryngeal vestibule closure: complete, partial, incomplete
Pharyngeal area at maximum constriction	PhAMPC	%(C2-4)^2^	Pixel-based measure of the 2-dimensional lateral area of the unobliterated pharynx on the frame of maximum pharyngeal constriction
Pharyngeal area at rest	PhAR	%(C2-4)^2^	Pixel-based measure of the 2-dimensional lateral area of the pharynx on the frame of swallow rest
Vallecular residue		%(C2-4)^2^	Pixel-based measure of the 2-dimensional lateral area of residue in the vallecular space, normalized to the squared length of the C2-4 vertebral spine
Pyriform sinus residue		%(C2-4)^2^	Pixel-based measure of the 2-dimensional lateral area of residue in the pyriform sinuses, normalized to the squared length of the C2-4 vertebral spine
Other pharyngeal residue		%(C2-4)^2^	Pixel-based measure of the 2-dimensional lateral area of residue elsewhere in the pharynx, normalized to the squared length of the C2-4 vertebral spine
Total pharyngeal residue		%(C2-4)^2^	Sum of residue measures for the valleculae, pyriform sinuses and elsewhere in the pharynx

To ensure accuracy and allow for the calculation of interrater reliability, each recording was rated in duplicate by two trained raters. Discrepancies across ratings were resolved by consensus in a review session involving three experienced raters. Interrater reliability was calculated based on ratings obtained prior to resolution. Image quality issues made it impossible to confidently identify peak hyoid position on a substantial number of the COPD videos; consequently, further analysis of this parameter was abandoned.

### Statistical Analysis

Statistical analyses were conducted using IBM SPSS Statistics Version 26 using a familywise *p* value of < .05. Descriptive statistics were calculated for each parameter by cohort and consistency. Reference range boundaries for each parameter of interest were taken from the Steele et al. (2019) supplementary data tables (https://steeleswallowinglab.ca/srrl/wp-content/uploads/ASPEKT-Method-Reference-Value-Tables-V1.3.pdf). As previously mentioned, at-risk values were defined as those falling > 1 *SD* from the reference mean for normally distributed parameters, and < Q1 or > Q3 for skewed parameters. Frequency tables for the number of participants showing at-risk parameter values were compiled and compared between the COPD and healthy cohorts by liquid consistency using either chi-square or Fisher's exact tests (depending on cell count) and odds ratios.

## Results

### Demographics

The COPD cohort was significantly older than the healthy reference group with a median age of 67 versus 30 years in the healthy reference group (Mann–Whitney *U* = 15, *p* < .001). The sex distribution of the COPD cohort was also more heavily weighted toward males (64%) compared to the sex-balanced reference cohort, although this difference was not statistically significant (χ^2^ = 1.6, *df* = 1, *p* = .21).

### Interrater Agreement and Descriptive Statistics

Absolute agreement between raters, prior to discrepancy resolution, was 88% for PAS scores; however, Fleiss kappa calculations showed low agreement (ĸ = .08). The standard operating procedure for resolving disagreements in initial PAS ratings ensured that consensus was achieved prior to carrying these ratings forward into the analysis. For ratings of LVC integrity (complete vs. incomplete), preconsensus absolute agreement was 83% with a Fleiss kappa score of .3. For the identification of the frame numbers for key events in the swallowing sequence, from which timing measures were derived, interrater agreement was excellent (intraclass correlation [ICC] = .997, 95% confidence interval [.996, .997]). Preresolution pixel-based measures of maximum pharyngeal constriction showed moderate interrater agreement with an ICC of .57 (95% confidence interval [.31, .73]). For measures of pharyngeal residue, good agreement was found with an ICC of .78 (95% confidence interval [.63, .87]). Lower values for interrater agreement are not unexpected for pixel-based measures that are derived based on measurement of two dimensional areas and reference scalars. All discrepant measures were reviewed in consensus meetings and remeasured or resolved, prior to proceeding with the statistical analysis. Descriptive statistics can be found by cohort and consistency in Tables 3 and 4 for parameters with normal and skewed distributions, respectively.

**Table 3.** Descriptive statistics by cohort and consistency for normally distributed parameters.

Parameter	Consistency	Healthy controls (*n* = 38)				COPD (*n* = 28)			
		*M*	*SD*	95% CI				95% CI	
				Lower bound	Upper bound	*M*	*SD*	Lower bound	Upper bound
Time-to-LVC (ms)	Thin	216	102	176	256	188	158	124	252
	Mildly thick	162	82	133	191	150	100	111	190
	Moderately thick	151	66	126	175	180	153	114	245
	Extremely thick	157	59	135	179	160	78	129	192
LVC duration (ms)	Thin	421	97	383	458	399	167	332	466
	Mildly thick	474	185	408	540	394	134	341	447
	Moderately thick	480	225	398	563	364	145	302	427
	Extremely thick	433	89	400	466	379	139	323	435
Hyoid burst to UES opening (ms)	Thin	104	59	81	127	83	60	58	107
	Mildly thick	129	58	109	150	88	59	64	111
	Moderately thick	147	51	129	166	126	111	78	175
	Extremely thick	151	57	129	172	111	49	91	131
UES opening duration (ms)	Thin	455	58	433	478	410	106	367	453
	Mildly thick	448	86	418	478	427	77	396	457
	Moderately thick	416	65	393	440	405	66	376	433
	Extremely thick	378	98	342	415	377	111	332	422
UES diameter, %(C2-4)	Thin	19	8	16	22	21	9	17	24
	Mildly thick	18	5	16	20	23	9	20	27
	Moderately thick	15	5	14	17	22	6	19	25
	Extremely thick	16	5	14	18	22	6	19	25
Pharyngeal area at rest, %(C2-4)^2^	Pooled across consistencies	57	17	55	60	65	16	58	72

*Note.* COPD = chronic obstructive pulmonary disease; CI = confidence interval; LVC = laryngeal vestibule closure; UES = upper esophageal sphincter.

**Table 4.** Descriptive statistics (percentiles) by cohort and consistency for parameters with skewed distributions.

Parameter	Consistency	Healthy controls (*n* = 38)			COPD (*n* = 28)		
		25th %ile	*Mdn*	75th %ile	25th %ile	*Mdn*	75th %ile
No. of swallows per bolus	Thin	1	1	1	1	1	2
	Mildly thick	1	1	1	1	1	2
	Moderately thick	1	1	1	1	1	2
	Extremely thick	1	1	1	1	1	2
Penetration–Aspiration Scale score	Thin	1	1	1	1	1	1
	Mildly thick	1	1	1	1	1	1
	Moderately thick	1	1	1	1	1	1
	Extremely thick	1	1	1	1	1	1
Swallow reaction time (ms)	Thin	33	67	167	83	181	264
	Mildly thick	0	56	217	83	167	361
	Moderately thick	0	67	667	56	139	250
	Extremely thick	0	50	709	79	194	459
Pharyngeal area at maximum constriction, %(C2-4)^2^	Thin	0	0	1	2	3	5
	Mildly thick	0	1	2	1	4	8
	Moderately thick	0	0	1	1	3	7
	Extremely thick	0	0	1	2	4	6
Vallecular residue, %(C2-4)^2^	Thin	0	0	0	0	0	1
	Mildly thick	0	0	1	0	0	2
	Moderately thick	0	0	0	0	0	1
	Extremely thick	0	0	0	0	0	1
Pyriform sinus residue, %(C2-4)^2^	Thin	0	0	0	0	0	0
	Mildly thick	0	0	0	0	0	1
	Moderately thick	0	0	0	0	0	0
	Extremely thick	0	0	0	0	0	0
Other residue, %(C2-4)^2^	Thin	0	0	0	0	0	0
	Mildly thick	0	0	0	0	0	0
	Moderately thick	0	0	0	0	0	0
	Extremely thick	0	0	0	0	0	0
Total pharyngeal residue, %(C2-4)^2^	Thin	0	0	0	0	0	2
	Mildly thick	0	0	1	0	1	4
	Moderately thick	0	0	0	0	0	2
	Extremely thick	0	0	0	0	0	2

*Note.* COPD = chronic obstructive pulmonary disease.

### Parameters Related to Swallowing Safety

**Figure 1.**
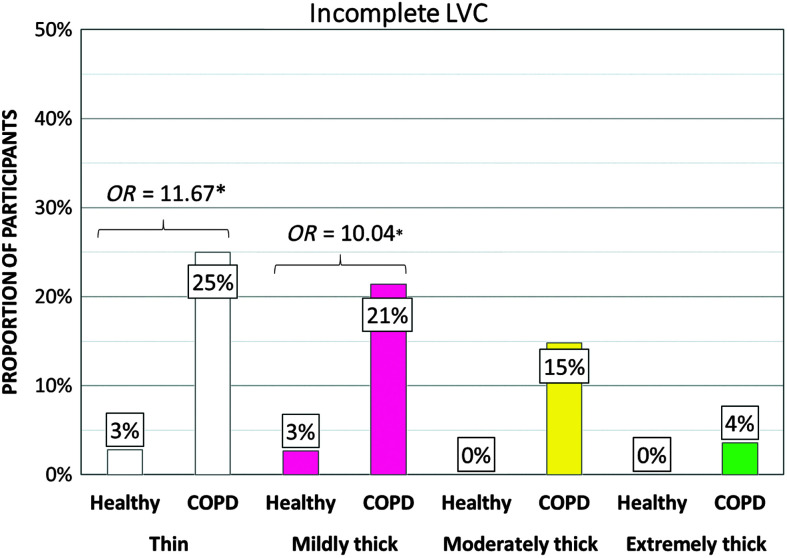
Frequencies (%) of participants with incomplete laryngeal vestibule closure (LVC) by group, with odds ratios (*OR*). COPD = chronic obstructive pulmonary disease.

**Figure 2.**
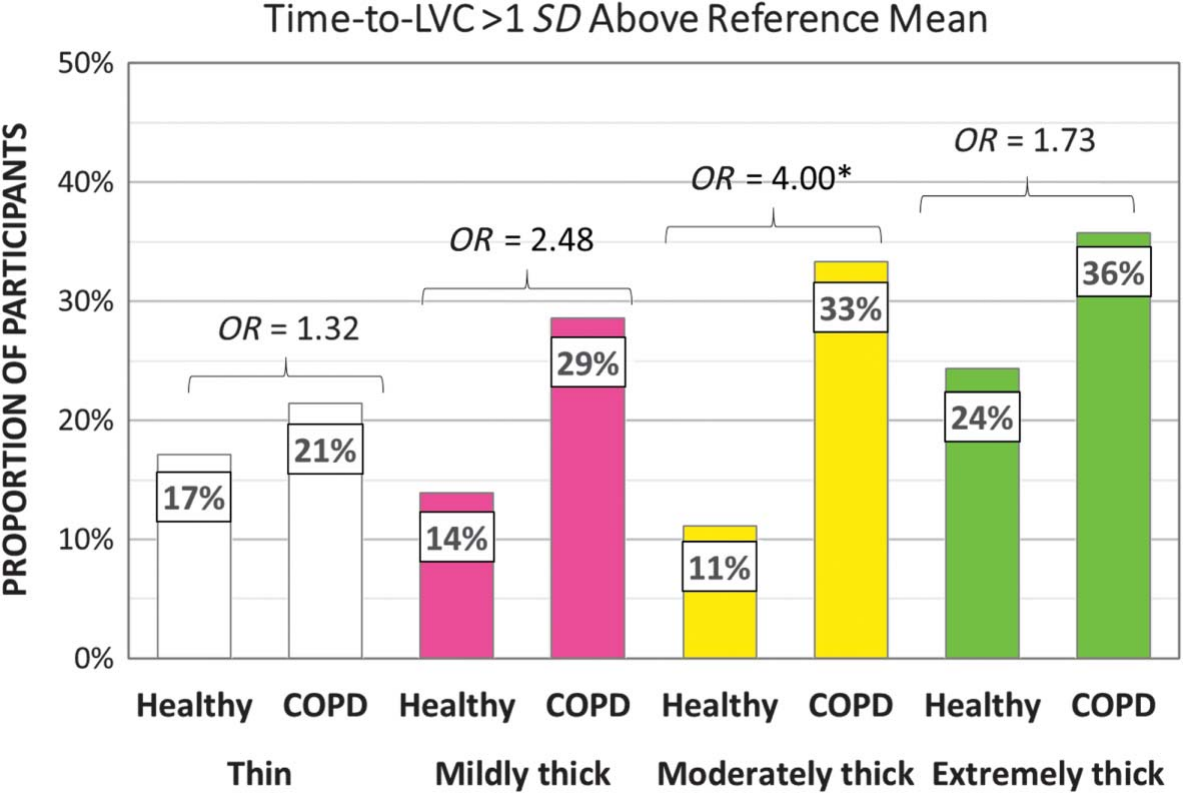
Frequencies (%) of participants with prolonged time-to-LVC (laryngeal vestibule closure), with odds ratios (*OR*). COPD = chronic obstructive pulmonary disease.

**Figure 3.**
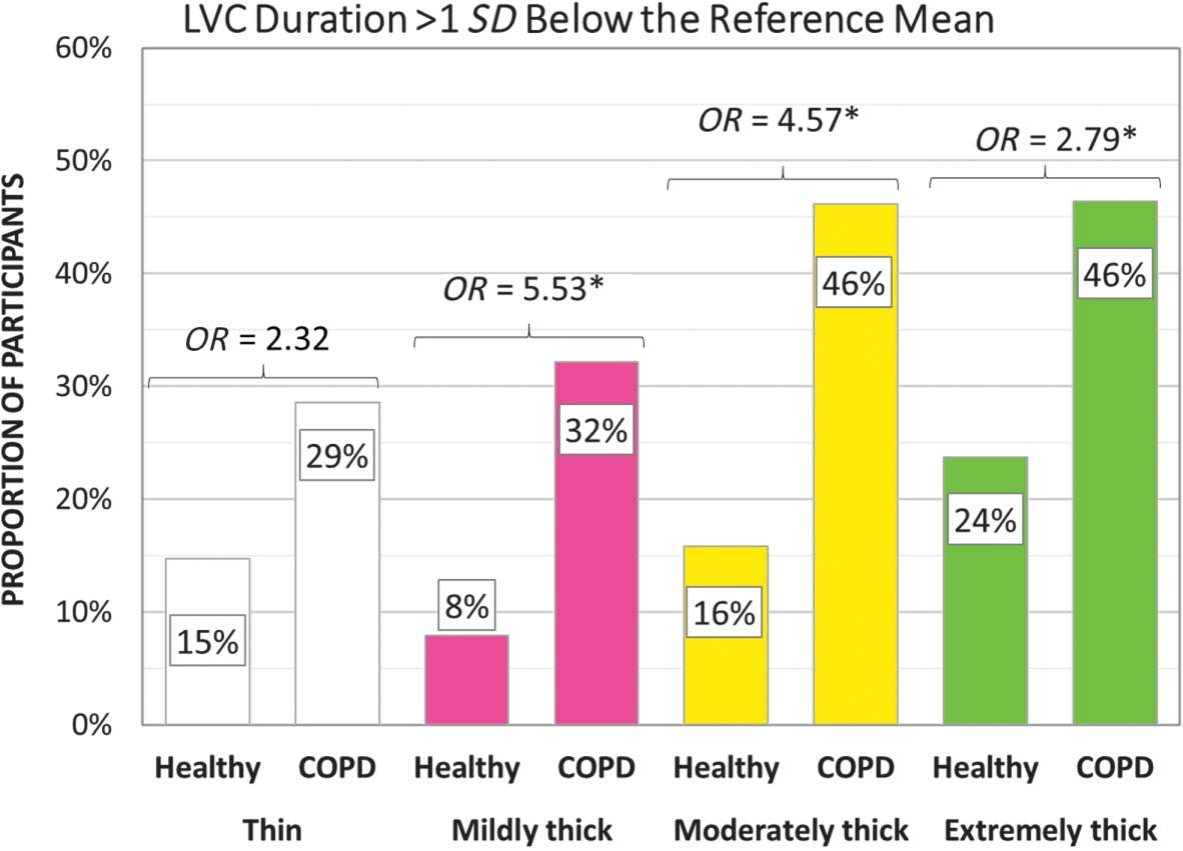
Frequencies (%) of participants with short laryngeal vestibule closure (LVC) duration by group, with odds ratios (*OR*s). COPD = chronic obstructive pulmonary disease.

Table 5 shows the frequencies of participants demonstrating at-risk values for parameters related to swallowing safety by cohort and consistency. PAS scores of 3 or higher turned out to be rare in the COPD cohort, and frequencies did not differ significantly from those seen in the healthy cohort. Notably, no penetration–aspiration events were seen in either group with the moderately and extremely thick consistencies. Despite the low frequencies of penetration or aspiration, the COPD cohort did display significantly higher frequencies of at-risk scores for parameters related to airway protection, including incomplete LVC (thin, mildly thick, and moderately thick liquids), long time-to-LVC (moderately thick liquids), and short LVC duration (mildly, moderately, and extremely thick liquids). Figures 1, 2, and 3 illustrate the odds ratios for at-risk scores related to LVC.

**Table 5.** Frequencies by group (*n*, %) of at-risk scores for parameters related to penetration–aspiration and laryngeal vestibule closure (LVC).

IDDSI level	Penetration–Aspiration Scale		LVC		Time-to-LVC		LVC duration	
	Score of 3 or worse		Incomplete		> 1 *SD* above reference mean		> 1 *SD* below reference mean	
	Healthy, *n* (%)	COPD, *n* (%)	Healthy, *n* (%)	COPD, *n* (%)	Healthy, *n* (%)	COPD, *n* (%)	Healthy, *n* (%)	COPD, *n* (%)
0 (thin)	1 (3)	1 (4)	1 (3)	7 (25)^a^	6 (17)	6 (21)	5 (15)	8 (29)
2 (mildly thick)	1 (3)	3 (11)	1 (3)	6 (21)^a^	5 (14)	8 (29)	3 (8)	9 (32)^a^
3 (moderately thick)	0 (0)	0 (0)	0 (0)	4 (15)^a^	4 (11)	9 (33)^a^	6 (16)	12 (46)^b^
4 (extremely thick)	0 (0)	0 (0)	0 (0)	1 (4)	9 (24)	10 (36)	9 (24)	13 (46)^a^

*Note.*  IDDSI = International Dysphagia Diet Standardisation Initiative; COPD = chronic obstructive pulmonary disease.

a Fisher's exact test showed significantly higher frequencies of at-risk scores in the COPD cohort, *p* < .05.

b Chi-square test showed significantly higher frequencies of at-risk scores in the COPD cohort, *p* < .01.

### Timing Parameters

**Figure 4.**
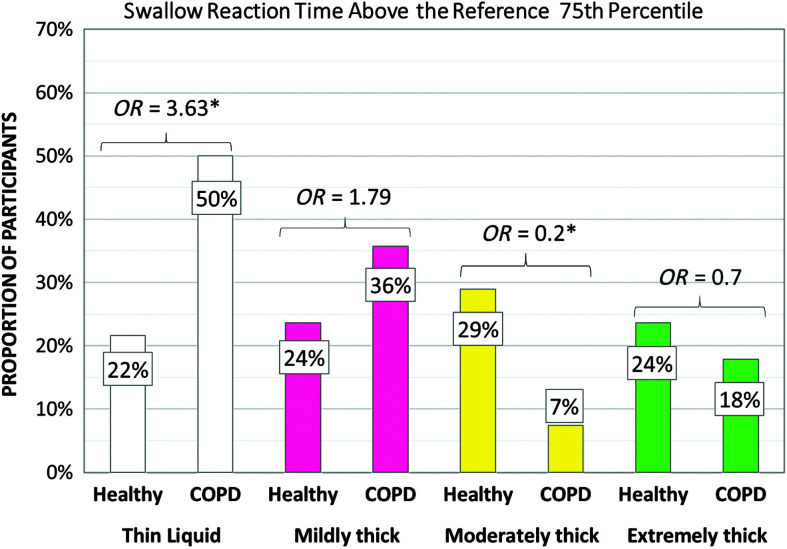
Frequencies (%) of participants with long swallow reaction time by group, with odds ratios (*OR*s). COPD = chronic obstructive pulmonary disease.

**Figure 5.**
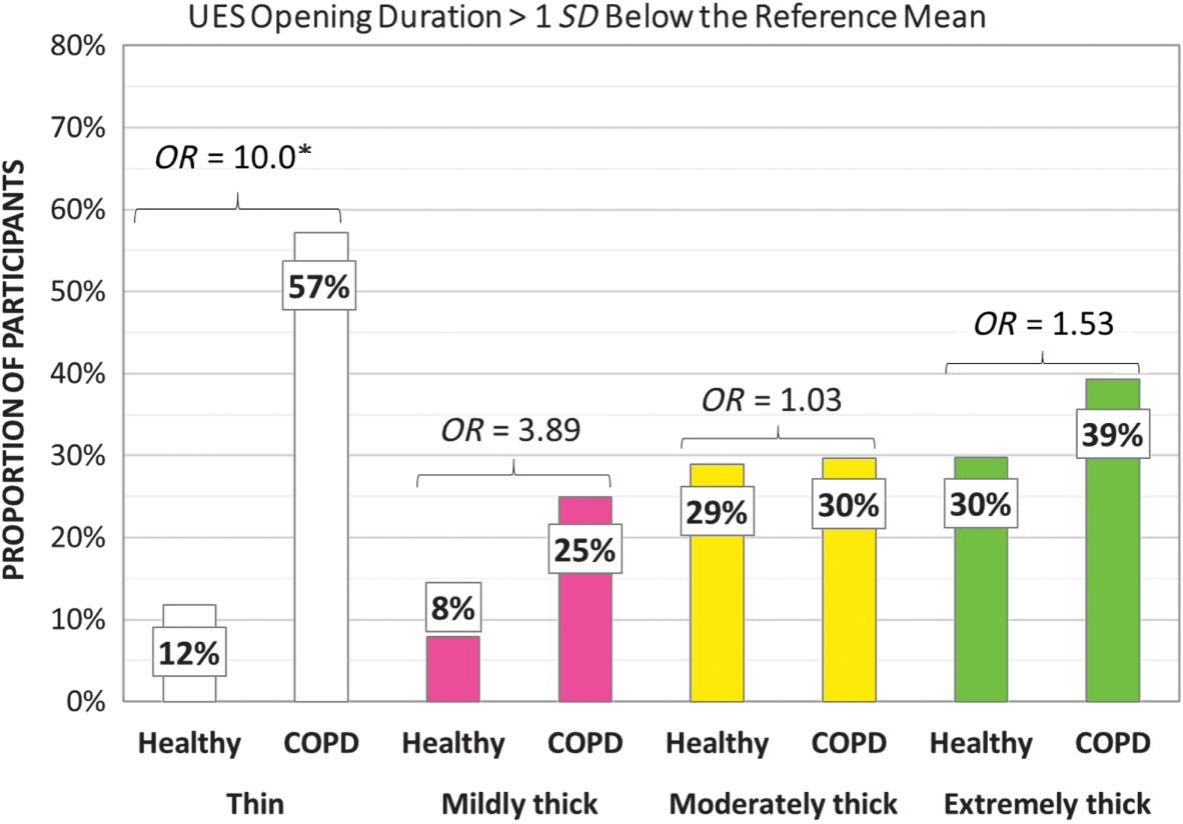
Frequencies (%) of participants with short upper esophageal sphincter (UES) opening duration by group, with odds ratios (*OR*s). COPD = chronic obstructive pulmonary disease.

**Figure 6.**
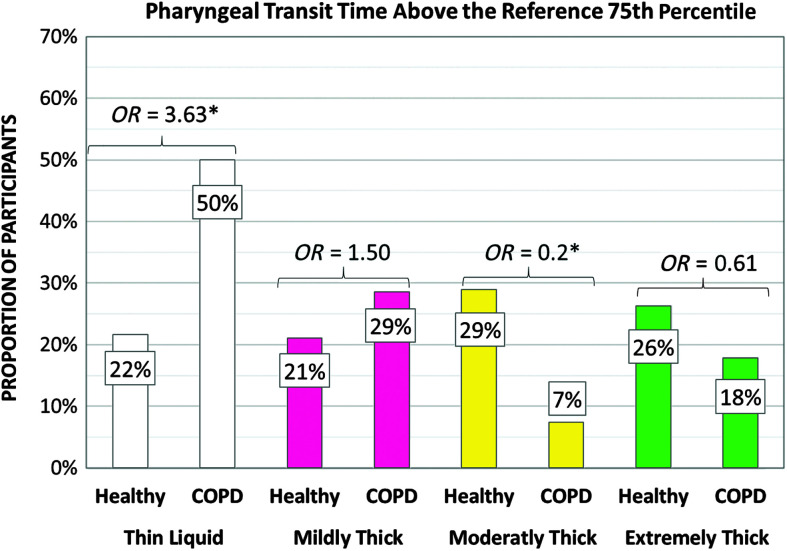
Frequencies (%) of participants with long pharyngeal transit time by group, with odds ratios (*OR*s). COPD = chronic obstructive pulmonary disease.

Table 6 shows the frequencies of participants demonstrating at-risk values for timing measures of swallowing by cohort and consistency. In the COPD cohort, significantly higher frequencies of at-risk values involving long swallow reaction times, short UES opening durations, and long pharyngeal transit times were seen with thin liquid. In the healthy cohort, an unexpected pattern of significantly higher frequencies of at-risk values was seen in the form of long swallow reaction times on moderately thick liquids, which contributed to longer pharyngeal transit time measures. Cohort differences in the frequencies of at-risk values were not seen for any timing parameters with mildly thick or extremely thick stimuli. Additionally, no cohort differences were found in the frequencies of at-risk values for measures of the hyoid burst to UES opening interval for any stimulus consistency or for measures of UES opening duration on mildly, moderately, and extremely thick stimuli. Figures 4 and 5 illustrate the odds ratios for at-risk swallow reaction time and UES opening duration. Figure 6 illustrates similar data for the composite measure of pharyngeal transit time, capturing the sum of swallow reaction time, the hyoid burst to UES opening interval, and UES opening duration.

**Table 6.** Frequencies by group (*n*, %) of at-risk scores for swallow timing measures.

IDDSI level	Swallow reaction time		HYB to UESO		UESO duration		Pharyngeal transit time	
	Above reference Q3		> 1 *SD* above reference mean		> 1 *SD* below reference mean		Above reference Q3	
	Healthy, *n* (%)	COPD, *n* (%)	Healthy, *n* (%)	COPD, *n* (%)	Healthy, *n* (%)	COPD, *n* (%)	Healthy, *n* (%)	COPD, *n* (%)
0 (thin)	8 (22)	14 (50)^a^	8 (22)	3 (11)	4 (12)	16 (57)^b^	8 (22)	14 (50)^a^
2 (mildly thick)	9 (24)	10 (36)	4 (11)	1 (4)	3 (8)	7 (25)	8 (21)	8 (29)
3 (moderately thick)	11 (29)	2 (7)^c^	2 (5))	2 (7)	11 (29)	8 (30)	11 (29)	2 (7)^c^
4 (extremely thick)	9 (24)	5 (18)	3 (8)	0 (0)	11 (30)	11 (39)	10 (26)	5 (18)

*Note.* IDDSI = International Dysphagia Diet Standardisation Initiative; HYB = hyoid burst; UESO = upper esophageal sphincter opening; Q3 = third quartile; COPD = chronic obstructive pulmonary disease.

a Chi-square test showed significantly higher frequencies of at-risk scores in the COPD cohort, *p* < .05.

b Fisher's exact test showed significantly higher frequencies of at-risk scores in the COPD cohort, *p* < .01.

c Fisher's exact test showed significantly higher frequencies of at-risk scores in the healthy cohort, *p* < .05.

### Pixel-Based Measures of Pharyngeal Residue

**Figure 7.**
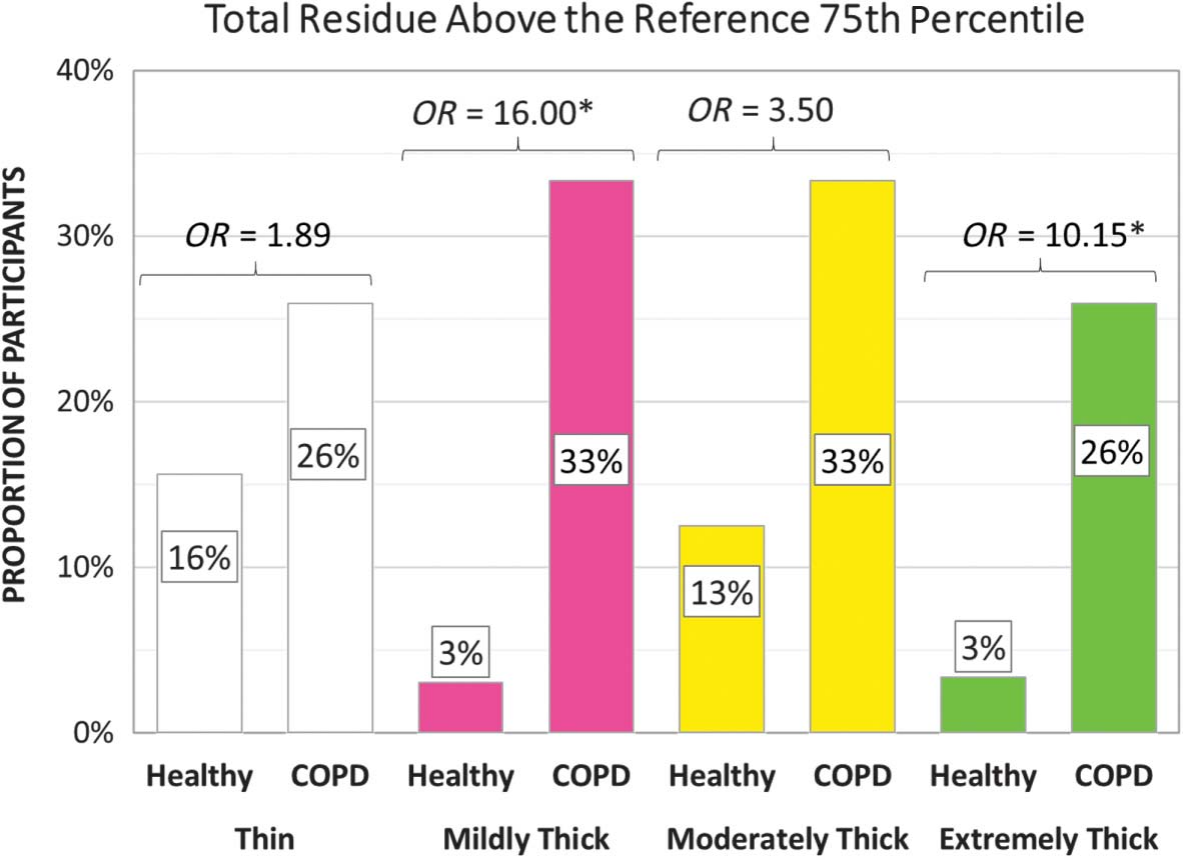
Frequencies (%) of participants with total pharyngeal residue measures of concern by group, with odds ratios (*OR*). COPD = chronic obstructive pulmonary disease.

Table 7 shows the frequencies of participants demonstrating at-risk values for pixel-based measures of residue in the valleculae, the pyriform sinuses, elsewhere in the pharynx, and overall (i.e., total pharyngeal residue) by cohort and consistency. There were no differences between cohorts in the frequency of at-risk residue with thin liquids and moderately thick liquids. However, at-risk residue with mildly thick liquids was significantly more common in the COPD cohort in the valleculae, pyriform sinuses, and overall, while at-risk residue with extremely thick liquids was more common in the COPD cohort for all locations. Figure 7 illustrates the odds ratios for total pharyngeal residue by cohort and consistency.

**Table 7.** Frequencies by group (*n*, %) of at-risk scores for measures of pharyngeal residue.

IDDSI level	Vallecular residue		Pyriform sinus residue		Other residue		Total pharyngeal residue	
	Above reference Q3		Above reference Q3		Above reference Q3		Above reference Q3	
	Healthy, *n* (%)	COPD, *n* (%)	Healthy, *n* (%)	COPD, *n* (%)	Healthy, *n* (%)	COPD, *n* (%)	Healthy, *n* (%)	COPD, *n* (%)
0 (thin)	6 (19)	9 (32)	3 (9)	7 (26)	1 (3)	2 (7)	5 (16)	7 (26)
2 (mildly thick)	3 (9)	10 (37)^a^	4 (12)	9 (33)^a^	2 (6)	6 (22)	1 (3)	9 (33)^b^
3 (moderately thick)	6 (19)	8 (33)	3 (9)	6 (24)	1 (3)	3 (12)	4 (13)	8 (33)
4 (extremely thick)	3 (10)	9 (33)^a^	0 (0)	8 (29)^b^	0 (0)	4 (14)^a^	1 (3)	7 (26)^a^

*Note.*  IDDSI = International Dysphagia Diet Standardisation Initiative; Q3 = third quartile; COPD = chronic obstructive pulmonary disease.

a Fisher's exact test showed significantly higher frequencies of at-risk scores in the COPD cohort, *p* < .05.

b Fisher's exact test showed significantly higher frequencies of at-risk scores in the COPD cohort, *p* < .01.

### Pixel-Based Measures of Structural Movement

**Figure 8.**
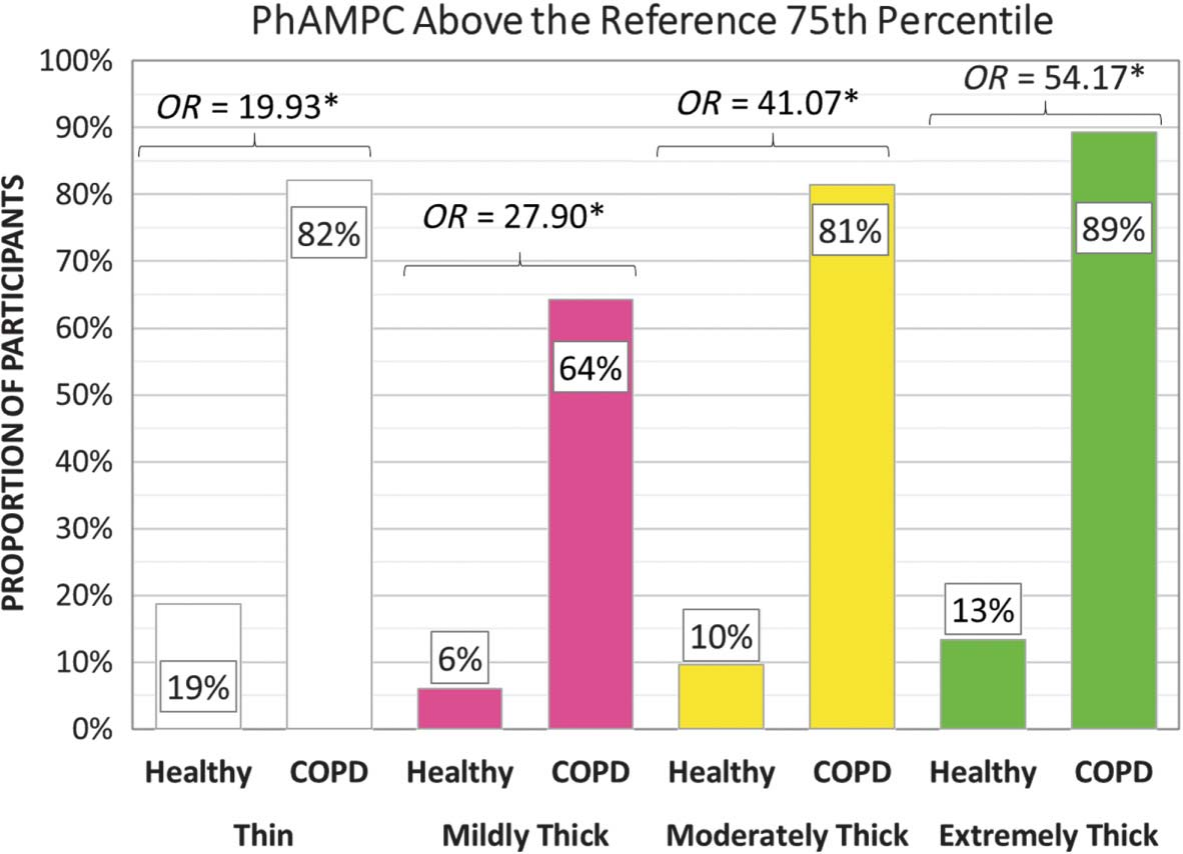
Frequencies (%) of participants with poor pharyngeal constriction by group, with odds ratios (*OR*s). COPD = chronic obstructive pulmonary disease; PhAMPC = pharyngeal area at maximum constriction.

As previously mentioned, image quality issues in the COPD data set resulted in a large number of missing data points for measures of peak XY hyoid position, and this parameter was not analyzed. Table 8 shows the frequencies of participants demonstrating at-risk values for pixel-based measures of pharyngeal area at rest and pharyngeal area at maximum constriction. There was no difference between cohorts in the frequency of enlarged pharyngeal area at rest. However, the COPD cohort showed significantly higher frequencies of poor pharyngeal constriction for all consistencies. This is illustrated in Figure 8.

**Table 8.** Frequencies by group (*n*, %) of at-risk scores for measures of pharyngeal constriction.

IDDSI level	Pharyngeal area at rest		Pharyngeal area at maximum constriction	
	Above reference Q3		Above reference Q3	
	Healthy, *n* (%)	COPD, *n* (%)	Healthy, *n* (%)	COPD, *n* (%)
0 (thin)	9 (25)^a^	5 (21)^a^	6 (19)	23 (82)^b^
2 (mildly thick)			2 (6)	18 (64)^c^
3 (moderately thick)			3 (10)	22 (82)^c^
4 (extremely thick)			4 (13)	25 (89)^c^

*Note.* IDDSI = International Dysphagia Diet Standardisation Initiative; Q3 = third quartile; COPD = chronic obstructive pulmonary disease.

a Measures of pharyngeal area at rest are not stratified by consistency.

b Chi-square test showed significantly higher frequencies of at-risk scores in the COPD cohort, *p* < .01.

c Fisher's exact test showed significantly higher frequencies of at-risk scores in the COPD cohort, *p* < .01.

## Discussion

The aim of this study was to analyze swallowing physiology in patients with stable COPD across the range from thin to extremely thick liquids in comparison to available reference data for healthy adults. We hypothesized that individuals with COPD would display greater frequencies of at-risk values for parameters associated with swallowing safety (i.e., penetration–aspiration and measures of LVC integrity and timing). We also expected to see higher frequencies of at-risk values in the COPD cohort for measures of swallow timing and swallowing efficiency, including pharyngeal constriction and pharyngeal residue.

The majority of the COPD cohort was classified as having disease severity at GOLD Level 3, which corresponds to severe airflow limitation with forced expiratory volume measures between 30% and 50% of predicted values (GOLD, 2020). Data from these individuals were compared to data from a previously collected, sex-balanced sample of healthy adults under 60 years of age. It is important to recognize that cohort differences in both age and sex distribution may explain some of the differences between groups.

### Swallowing Safety and LVC

This cohort of COPD patients did not display significantly higher frequencies of penetration or aspiration than the healthy reference values study sample (Steele et al., 2019). None of the participants in the COPD cohort aspirated material below the level of the vocal folds (i.e., PAS ≥ 6). We speculate that this finding may reflect the fact that our participants had stable COPD rather than being in exacerbation. The prevalence of penetration–aspiration seen in our COPD cohort is lower than that described by Garand et al. (2018), who observed a maximum PAS score of 8 in their COPD patients, indicating silent aspiration, and a maximum PAS score of 5, indicating penetration in their healthy control group. One reason for the discrepancy in findings across studies may relate to the videofluoroscopy protocols used. In our study, we examined single sips or spoons-full of each consistency, whereas Garand et al.'s study involved the MBSImP protocol, which captures worst scores across several different volumes of each consistency tested, including a large-volume sequential drinking task, which has been shown to elicit penetration–aspiration more frequently than smaller volume tasks (Hazelwood et al., 2017; Leder et al., 2011; Martin-Harris et al., 2008).

Despite the absence of penetration–aspiration, the COPD cohort in our study demonstrated significantly higher frequencies of at-risk values for all three parameters measuring aspects of LVC. Previous studies suggest that the risk of penetration and aspiration is much higher in situations of incomplete LVC (Steele et al., 2015; Vose & Humbert, 2018). Prolonged time-to-LVC, previously called “LVC reaction time” (Humbert et al., 2018; Steele et al., 2019), is also thought to be a characteristic that increases the risk of penetration and aspiration (Nativ-Zeltzer et al., 2014; Steele et al., 2015). Longer time-to-LVC has been found to differentiate between PAS scores of 1 and 2 in a sample of healthy older adults recently reported by Herzberg et al. (2019). Due to the lack of age-matched controls in this study, we are unable to determine whether the prolonged time-to-LVC values seen in the COPD cohort exceed values that would be expected in healthy adults of a similar age. Herzberg et al. (2019) have also described shorter time-to-LVC in older adults with more advanced bolus location at swallow onset and interpreted this to represent a spontaneous compensation designed to facilitate airway protection. Bolus location at swallow onset was not analyzed in our study; therefore, it remains unknown whether this compensatory phenomenon was present or absent in the COPD cohort.

An interesting finding in this study is the higher prevalence of short LVC durations in the COPD cohort. This contrasts with a previous study by Cassiani et al. (2015), who reported significantly longer LVC durations on 5-ml thin liquid boluses in a group of 16 patients with COPD compared to healthy controls. The reasons for the discrepancy in findings are not clear but may include differences in participant disease severity and study methodology (e.g., a much more concentrated barium product and bolus delivery by syringe). Previous studies in patients with stroke, amyotrophic lateral sclerosis, oropharyngeal cancer treated with radiation, and oculopharyngeal muscular dystrophy have failed to find differences in LVC duration between individuals with versus without penetration and aspiration (Barbon et al., 2019; Park et al., 2010; Waito et al., 2020, 2018).

One possible explanation for short LVC duration in individuals with COPD relates to respiratory–swallow coordination. Individuals with COPD are more likely to display abnormal respiratory–swallow coordination patterns involving inhalation immediately before or after the swallow (Cvejic et al., 2011; Nagami et al., 2017). Additionally, a recent treatment study in patients with head and neck cancer, of whom 37% had COPD, suggests that respiratory–swallow discoordination may also involve initiation of swallowing later in the respiratory cycle (Martin-Harris et al., 2016). It is tempting to speculate that the shorter durations of LVC seen in the COPD cohort might reflect some aspect of disordered respiratory–swallow coordination and an urgency to resume breathing in the context of pulmonary hyperinflation and reduced expiratory volumes. However, given that the respiratory pause in swallowing is under neural control and not necessarily time-linked to mechanical closure of the laryngeal vestibule, simultaneous measures of airflow or chest wall movement with videofluoroscopy would be needed to properly explore this hypothesis in future studies.

### Timing Measures

Timing differences were seen in the COPD cohort in the form of increased odds of prolonged swallow reaction times on thin and mildly thick liquids, which, in turn, appear to explain associated findings of longer pharyngeal transit times on these stimuli. Long latencies to swallow initiation can reflect either sensory and/or motor deficits. To our knowledge, there is no reason to suspect that individuals with COPD would experience motor difficulties with swallow onset. However, impairments in laryngeal sensation have been reported in the COPD population (Clayton et al., 2014), with one possible contributing factor being the high use of inhaled corticosteroid medications in this population. Whether sensory deficits also exist in the oropharynx and manifest in the form of long swallow reaction times remains less clear (Borowsky da Rosa et al., 2020). Interestingly, the current study actually found higher rates of prolonged swallow reaction times on moderately and extremely thick liquids in the healthy reference sample. The reasons for this pattern remain unclear, given the fact that the moderately thick boluses in both studies had matched IDDSI Flow Test results of 9.6 ml. Differences in sip size cannot be excluded as a contributing factor, given that the COPD data represent sips from a cup whereas the moderately thick stimuli in the reference study were taken by 5-ml teaspoon.

Longer pharyngeal transit times have been reported as a feature of swallowing in individuals with COPD in previous studies (Cassiani et al., 2015; Chaves et al., 2014). In this study, higher odds of prolonged pharyngeal transit time in those with COPD were only seen for thin and mildly thick liquids. Caution is warranted when comparing this result to prior studies, given that definitions of the pharyngeal transit time measure differ across studies (Namasivayam-MacDonald et al., 2018). Differences in barium concentration, bolus volume, methods of bolus administration, and the use (or not) of swallow cues may also contribute to discordant results across studies.

The final timing difference seen in the COPD cohort was a higher prevalence of short UES opening duration, particularly with thin liquid stimuli. It is possible that this phenomenon is associated with the short durations of LVC seen in the COPD cohort, which we speculate may reflect reduced tolerance of apnea and an urgency to breathe. As previously mentioned, there is also some suggestion in the literature that chronic recruitment of accessory respiratory muscles in people with COPD results both in shorter breathing cycles (Dourado et al., 2006) and in potential tethering of the hyolaryngeal complex (Steidl et al., 2015; Yawn, 2013), which is biomechanically connected to the UES (Mendelsohn & McConnel, 1987).

### Pharyngeal Constriction and Pharyngeal Residue

Our second hypothesis, which was based on evidence in the literature that individuals with COPD may present with muscle weakness, predicted a higher frequency of at-risk values for peak hyoid position, pharyngeal constriction, and pharyngeal residue. This hypothesis was confirmed with the exception of the hyoid measures, which we were unable to complete.

**Figure 9.**
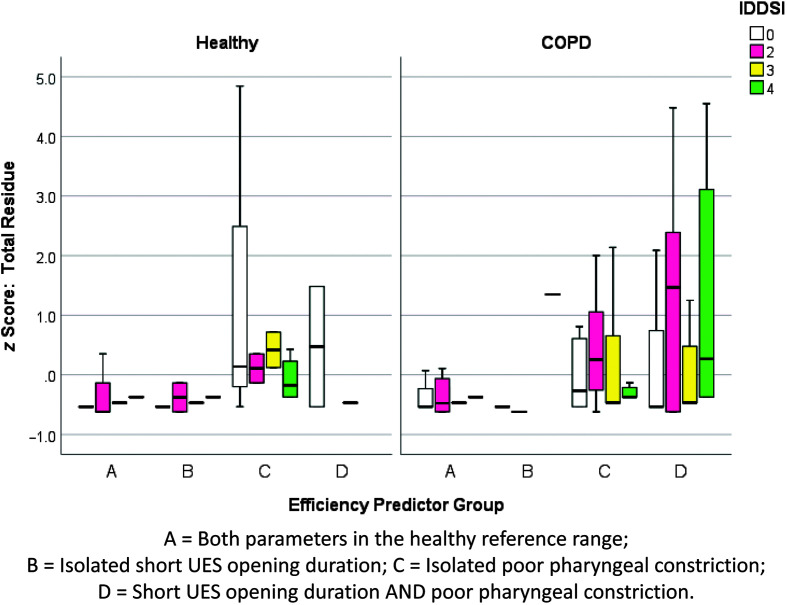
Box plots for *z* scores of total pharyngeal residue, by consistency, by group, according to efficiency predictor categories. COPD = chronic obstructive pulmonary disease; IDDSI = International Dysphagia Diet Standardisation Initiative; UES = upper esophageal sphincter.

Figure 8 illustrates one of the most dramatic findings in this study, in the form of markedly higher odds of reduced pharyngeal constriction in the COPD cohort for all consistencies. Higher odds of pharyngeal residue were also found in the COPD cohort for all consistencies in all locations, although significant group differences in the frequencies of at-risk residue were only seen with the mildly thick and extremely thick stimuli. To our knowledge, prior studies have not highlighted poor pharyngeal constriction as a characteristic of swallowing in COPD (e.g., Cassiani et al., 2015; Chaves et al., 2014; Garand et al., 2018). In other populations, reduced pharyngeal constriction has been identified as a predictor of residue (Curtis et al., 2020; Leonard et al., 2011; Stokely et al., 2015; Waito et al., 2020, 2018). Other potential predictors of residue include reduced pharyngeal shortening, which could arise in COPD due to antagonistic contraction of accessory respiratory muscles (Steidl et al., 2015; Yawn, 2013), and reduced diameters or durations of UES opening (Dejaeger et al., 1997; Kahrilas et al., 1988). Given the possibility that people with COPD might present with multiple predictors of residue, we decided to conduct post hoc analysis of the independent and joint contributions of at-risk pharyngeal constriction and UES opening durations to residue in our COPD cohort. First, the pixel-based measures of total pharyngeal residue were transformed into *z* scores within each consistency, across the entire sample, including COPD and healthy participants. Participants were classified into four “efficiency predictor” groups, capturing whether their values for pharyngeal area at maximum constriction and UES opening duration on each consistency were (a) both within the reference range, (b) showed isolated at-risk UES opening duration, (c) showed isolated at-risk pharyngeal constriction, or (d) showed both at-risk pharyngeal constriction and at-risk UES opening duration. A box plot was then generated, showing the relationship between total pharyngeal residue severity (by *z* score) and efficiency predictor group by consistency (see Figure 9). The results suggest that at-risk pharyngeal constriction is an independent and, by far, the strongest predictor of pharyngeal residue in the healthy cohort. Combined short UES opening and reduced pharyngeal constriction was rarely seen in the healthy reference group. By contrast, in the COPD cohort, the coexistence of short UES opening and poor pharyngeal constriction was both common and strongly predictive of residue that was worse than that seen with poor pharyngeal constriction alone.

### Older Age as an Explanation for Findings in the COPD Group

Given the acknowledged age difference between the two cohorts, we performed an exploratory post hoc analysis of the COPD data to evaluate the possibility that older age might be contributing to the higher frequencies of at-risk values observed in the COPD cohort. Spearman correlations were calculated for age and all continuous variables both overall and within consistency. Older age in the COPD cohort was found to be correlated (*r_s_* ≥ .28) with several parameters, as follows:

- shorter LVC durations on extremely thick liquids (*r_s_* = −.39, *p* = .04);
- shorter UES opening durations overall (*r_s_* = −.28, *p* < .01);
- smaller UES opening diameter on thin and mildly thick liquids in the COPD cohort, with correlations of *r_s_* = −.35 (*p* = .07) and *r_s_* = −.39 (*p* = .04), respectively;
- greater pyriform sinus residue on moderately and extremely thick liquids in the COPD cohort, with correlations of *r_s_* = −.5 (*df* = 28, *p* = .01) and *r_s_* = −.3 (*df* = 28, *p* = .12), respectively;
- greater total pharyngeal residue on extremely thick liquids, *r_s_* = −.32 (*df* = 27, *p* = .1).

Given the absence of age-matched healthy control data and reference data for older adults, it will be important for future studies to elucidate whether the magnitude and direction of these differences are similar to changes seen in presbyphagia or whether they represent features that are specific to individuals with COPD. Interestingly, despite older age, the COPD cohort did not display significant increases in measures of pharyngeal area at rest, which have been noted as a characteristic of aging and attributed to age-related muscle atrophy in other studies (Molfenter et al., 2019). One reason for the absence of obvious age-related changes to pharyngeal area in this study may be the fact that the COPD cohort did not include any participants over 79 years of age.

### Limitations

As with any study, several limitations must be noted. First, as noted in the discussion, the COPD cohort was older than the reference group, such that some of the observed differences may be explained by age rather than diagnosis. It is also acknowledged that, although the stimuli used in the COPD and the reference cohorts were matched for flow characteristics using the IDDSI Flow Test (Hanson et al., 2019) and were thickened with the same xanthan gum thickening agent, the barium products used were different. Similarly, differences in methods of bolus administration existed across the two data collection protocols; the relevance of differences in sip volume for timing and kinematic measures of swallowing is not fully understood at this time.

The analysis in this study involved comparisons of the frequency of at-risk values for a long list of parameters related to swallowing safety, timing, and efficiency. We acknowledge that a familywise *p*-value criterion of < .05 was used for all comparisons, within consistency, without adjustment for the repeated-measures nature of exploring four different consistencies or for the possible nonindependence of results across correlated parameters.

The fact that 28.9% of the healthy reference sample (i.e., 11/38 participants) displayed at-risk values for swallow reaction time may, at first glance, raise questions, given that the Q3 reference threshold came from the same individuals, implying that not more than 25% of them (i.e., 10 participants) should have measures above the threshold. The discrepancy probably reflects the fact that the current study data were derived from the first bolus of each consistency from the reference sample, whereas the published reference thresholds were determined based on three repeated boluses per consistency.

### Clinical Implications

In this study, we sought to characterize the physiology of swallowing in a group of stable COPD patients who underwent a videofluoroscopic swallowing examination. We hypothesized that, even if patients did not display penetration–aspiration, a detailed analysis might reveal altered swallowing physiology that might place a person at increased risk of airway invasion. The study results revealed impairments of the integrity, timing, and duration of LVC as risk factors in this regard. It is important to note that the patients with COPD in our study were stable and that swallowing function may differ between COPD patients who are stable versus those with exacerbation (Kobayashi et al., 2007; Terada et al., 2010). We know that adequate protective reflexes in the airway play an important role in the prevention of aspiration of oropharyngeal or gastric secretions that are colonized with bacteria. Impairment of these reflexes represents a potential risk factor for exacerbations of COPD (Kobayashi et al., 2007; Terada et al., 2010). For a complete picture, future studies in people who are experiencing COPD exacerbation should explore swallowing physiology, respiratory–swallow coordination, and cough function (Troche et al., 2014).

The observation of poor pharyngeal constriction in our COPD cohort for all consistencies highlights impaired swallowing efficiency, which has received little attention in previous studies. The odds ratios for residue-of-concern were particularly high on thicker consistencies in our study cohort, suggesting that texture modification should not be implemented in people with COPD without proper evaluation. The combination of poor pharyngeal constriction and short durations of UES opening represents a particular risk for postswallow residue, which may represent a further risk for subsequent aspiration. There is a need to conduct future research to determine the reasons for poor pharyngeal constriction in people with COPD and to develop effective interventions to prevent or remediate this concern. Similarly, it is important to explore whether treatment strategies focusing on respiratory–swallow coordination, which have recently been shown to be effective for adjusting the timing of swallowing within the respiratory cycle (Martin-Harris et al., 2016), also have beneficial impact on the duration of UES opening.

## Conclusions

In summary, this study reveals differences in swallowing physiology in patients with stable COPD. Differences in the integrity, timing, and duration of LVC represent a heightened risk for penetration–aspiration, while poor pharyngeal constriction and shorter UES opening represent risks for pharyngeal residue. Future studies are needed to understand how these aspects of swallowing change during COPD exacerbation. Future studies involving simultaneous measurement of respiratory and swallowing behaviors are highly recommended to elucidate the impact of respiratory–swallow coordination on swallowing function and physiology.

## Author Contributions

**Renata Mancopes:** Conceptualization (Lead), Data curation (Lead), Formal analysis (Equal), Investigation (Equal), Methodology (Equal), Resources (Equal), Validation (Equal), Writing – original draft (Lead), Writing – review & editing (Equal). **Melanie Peladeau-Pigeon:** Data curation (Equal), Methodology (Supporting), Project administration (Lead), Software (Lead), Writing – review & editing (Supporting). **Emily Barrett:** Data curation (Supporting), Resources (Supporting), Writing – eeview & editing (Supporting). **Andrea Guran:** Data curation (Supporting), Resources (Supporting), Writing– review & editing (Supporting). **Sana Smaoui:** Data curation (Supporting), Resources (Supporting), Writing– review & editing (Supporting). **Adriane Schmidt Pasqualoto:** Conceptualization (Supporting), Data curation (Equal), Resources (Supporting), Writing – review & editing (Supporting). **Catriona M. Steele:** Data curation (Equal), Formal analysis (Equal), Investigation (Equal), Methodology (Lead), Project administration (Equal), Supervision (Lead), Validation (Lead), Writing – review & editing (Lead).
